# 20-Hydroxycholecalciferol, Product of Vitamin D3 Hydroxylation by P450scc, Decreases NF-κB Activity by Increasing IκBα Levels in Human Keratinocytes

**DOI:** 10.1371/journal.pone.0005988

**Published:** 2009-06-19

**Authors:** Zorica Janjetovic, Michal A. Zmijewski, Robert C. Tuckey, Damon A. DeLeon, Minh N. Nguyen, Lawrence M. Pfeffer, Andrzej T. Slominski

**Affiliations:** 1 Department of Pathology and Laboratory Medicine, the Center for Cancer Research, University of Tennessee Health Science Center, Memphis, Tennessee, United States of America; 2 School of Biomedical, Biomolecular and Chemical Sciences, University of Western Australia, Crawley, Australia; Ludwig-Maximilians-University, Germany

## Abstract

The side chain of vitamin D3 is hydroxylated in a sequential manner by cytochrome P450scc (CYP11A1) to form 20-hydroxycholecalciferol, which can induce growth arrest and differentiation of both primary and immortalized epidermal keratinocytes. Since nuclear factor-κB (NF-κB) plays a pivotal role in the regulation of cell proliferation, differentiation and apoptosis, we examined the capability of 20-hydroxycholecalciferol to modulate the activity of NF-κB, using 1,25-dihydroxycholecalciferol (calcitriol) as a positive control. 20-hydroxycholecalciferol inhibits the activation of NFκB DNA binding activity as well as NF-κB-driven reporter gene activity in keratinocytes. Also, 20-hydroxycholecalciferol induced significant increases in the mRNA and protein levels of the NF-κB inhibitor protein, IκBα, in a time dependent manner, while no changes in total NF-κB-p65 mRNA or protein levels were observed. Another measure of NF-κB activity, p65 translocation from the cytoplasm into the nucleus was also inhibited in extracts of 20-hydroxycholecalciferol treated keratinocytes. Increased IκBα was concomitantly observed in cytosolic extracts of 20-hydroxycholecalciferol treated keratinocytes, as determined by immunoblotting and immunofluorescent staining. In keratinocytes lacking vitamin D receptor (VDR), 20-hydroxycholecalciferol did not affect IκBα mRNA levels, indicating that it requires VDR for its action on NF-κB activity. Comparison of the effects of calcitrol, hormonally active form of vitamin D3, with 20-hydrocholecalciferol show that both agents have a similar potency in inhibiting NF-κB. Since NF-κB is a major transcription factor for the induction of inflammatory mediators, our findings indicate that 20-hydroxycholecalciferol may be an effective therapeutic agent for inflammatory and hyperproliferative skin diseases.

## Introduction

**Figure 1:**
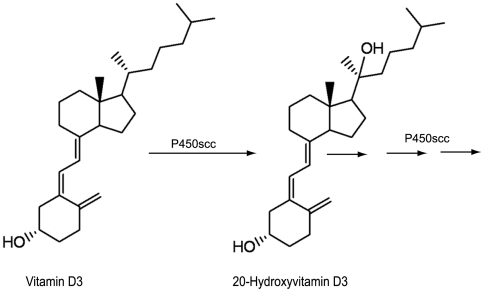
P450scc (CYP11A1) can hydroxylate vitamin D3 to 20-hydroxycholecalciferol with following sequential metabolism to other hydroxyderivatives [10].

Human keratinocytes have the ability to metabolize vitamin D3 autonomously [1], [2], [3]. Not only are they the site of conversion of 7-dehydrocholesterol (7DHC) to vitamin D3 following UVB-radiation [2], [4], [5], but they also express enzymes to hydroxylate vitamin D3 to the hormonally active form known as calcitriol or 1,25-dihydroxyvitamin D3 (1,25(OH)_2_D3). Thus, keratinocytes are the site of production and the target for vitamin D3 [3]. We have recently identified new pathway for metabolism of vitamin D and pro-vitamin D that is catalyzed by cytochrome P450scc (CYP11A1) [6], [7], [8], [9], [10], [11], [12], the enzyme catalyzing the conversion of cholesterol to pregnenolone for steroid hormone synthesis [13]. 20-Hydroxyvitamin D3 (20(OH)D3) is the major product of P450scc activation of vitamin D3 as well as an intermediate in the sequential synthesis of other hydroxylated derivatives including 20,23-dihydroxyvitamin D3 (20,23(OH)2D3 and 17,20,23-trihydroxyvitamin D3 (17,20,23(OH)3D3) (see Figure 1) [7], [10], [11]. We postulated that 20(OH)D3 could have systemic effects when produced in organs expressing high levels of P450scc, such as adrenal cortex, corpus luteum, follicles and placenta [7], [14], while in organs expressing low levels of P450scc, such as skin [8], 20(OH)D3 could serve local paracrine, autocrine or intracrine roles. In fact, we have recently demonstrated that 20(OH)D3 can stimulate differentiation and inhibit proliferation of keratinocyte cultured in vitro [15].

Vitamin D3 has a wide variety of actions in autoimmune diseases and cancer [16], [17], [18], [19], [20] as well as on bone physiology and blood pressure [21], [22], [23]. The biological role of its metabolite 20(OH)D3 is only partially known [15], while it is well documented that 1,25(OH)_2_D3 and its derivatives [24] have diverse biological activities on multiple cell lineages [2], [21], [25], [26], [27], including modulation of the skin immune system (SIS) and protection of the skin against UVB-induced damage [25], [26], [28], [29], [30], [31], [32].

Inflammation and proliferation are regulated by a plethora of transcription factors, with nuclear factor-κB (NF-κB) considered to be a master regulator of these processes (reviewed in [33]). NF-κB is also important in the development, prevention and therapy of cancer [34], [35], [36]. NF-κB activity is stimulated by many pathways that converge on IκB kinases, including the signaling pathways activated by various cytokines, such as the proinflammatory cytokine IL-1 (reviewed in [37], [38]), lipopolysaccharide (LPS) and tumor necrosis factor α (TNF-α) [39], [40]. In mammals, the NF-κB family of proteins includes NF-κB1 (p105 processed to p50), NF-κB2 (p100 processed to p52), RelA (p65), RelB and cRel [41]. Phosphorylation and subsequent degradation of IκB proteins allow for translocation of cytoplasmic NF-κB into the nucleus, where NF-κB binds to specific promoter/enhancer elements to regulate the expression of specific genes [33]. NF-κB regulated genes play important roles in inflammation, immunity, cell growth and cell survival [42], [43], [44], [45].

NF-κB activation is mediated through the activation of specific IκB kinases (IKKs) and the subsequent phosphorylation of IκB. The pathway leading to proteolysis of IκB is denoted as the canonical NF-κB activation pathway. NF-κB activation also occurs through the ‘noncanonical’ pathway, which does not involve IκB degradation and is activated by various agents, including interferon-α/β, lipopolysaccharide, the LMP1 protein of Epstein-Barr virus, B-cell activating factor and lymphotoxin-β [42], [46].

Vitamin D and various synthetic vitamin D analogues have been widely used in the treatment of psoriasis [47] and other inflammatory/hyperproliferative skin disorders [48], [49]. The cellular actions of 1,25(OH)_2_D3, the bioactive form of vitamin D, are not fully understood, but its effects have traditionally been ascribed to its binding to the vitamin D receptor (VDR) [50], [51], [52], [53]. NF-κB plays an important role in protecting keratinocytes against apoptosis during programmed cornification [54]. In normal human keratinocytes, 1,25(OH)_2_D3 reduces NF-κB DNA binding activity by increasing IκBα protein levels, which inhibits IL-8 production [55]. A similar effect is also seen in murine macrophages [56], [57]. Effects of 1,25(OH)_2_D3 on NF-κB that are not mediated by the VDR have also been reported for fibroblasts lacking the VDR [58].

In the present study we have examined the effects of 20(OH)D3 on NF-κB signaling in comparison to well defined effects of 1,25(OH)_2_D3. Since NF-κB dysregulation induces malignant transformation of HaCaT keratinocytes, but not of normal keratinocytes [59], we used in these studies both immortalized human HaCaT keratinocytes and primary epidermal keratinocytes, isolated from human neonatal foreskin (HEKn). The effects of 1,25(OH)_2_D3 on both expression of genes involved in its metabolism and the biological activity of the encoded proteins have previously been studied in these cells [26], [60]. Recent data from our laboratories indicates that 20(OH)D3 can be produced by adrenal mitochondria [7], that adrenal glands ex-vivo can transform 7DHC to 5,7-diene products [61] that in the skin can be converted to biologically active vitamin D-like products [62]. Therefore, action of 20(OH)D3 on NF-κB activity would suggest a role for a novel endogenous secosteroidogenic metabolic pathway [7], [8], [9], [10], [11] in the regulation of the systemic and cutaneous immune activity.

## Results

### 20-Hydroxycholecalciferol inhibits NF-κB DNA binding activity in keratinocytes

**Figure 2:**
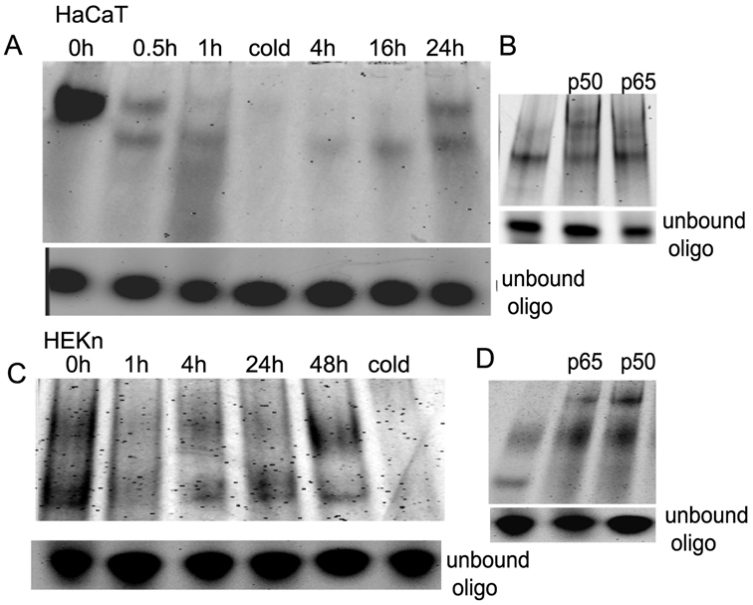
20(OH)D3 treatment inhibits the activation of NFκB DNA binding activity in keratinocytes. Nuclear extracts were prepared from human keratinocytes, normal and HaCaT, treated with 100 nM 20(OH)D3 for indicated time periods, and were subjected to EMSA (A,C). Nuclear extracts were preincubated with NF-κB antibodies (p65 and p50) and subjected to supershift assay (B,D). “Cold” represents nuclear extract preincubated with an excess of unlabelled oligonucleotide.

In initial experiments we determined that 20(OH)D3 at 100 nM was optimal for inducing biological actions like stimulation of keratinocytes differentiation and inhibition of cell proliferation [15]. Next we examined the effect of 20(OH)D3 on NF-κB activity in keratinocytes by assaying nuclear extracts of 20(OH)D3-stimulated cells by DNA-binding assays. Primary human keratinocytes and HaCaT cells were incubated with 100 nM 20(OH)D3, nuclear extracts were prepared and incubated with an NF-κB oligonucleotide probe based on the κB binding site in the immunoglobulin light chain enhancer. As shown in figure 2A and 2C, a time dependent decrease in nuclear protein binding to the κB response element was observed in extracts from 20(OH)D3-treated cells. Inhibition of NF-κB activity was observed within 30 minutes of 20(OH)D3 addition. Maximum inhibition was reached by 4 hours, and inhibition persisted up to 24 hours. This effect was no longer observed after 48 hours. Treatment with 1,25(OH)_2_D3 also had similar inhibitory effect of NF-κB activity (data not shown). High basal NF-κB activity in HaCaT cells is probably due to serum deprivation of cells, since previously we have demonstrated that serum deprivation triggers NF-κB activation in HaCaT cells [63].

In order to determine the composition of 20(OH)D3-induced NF-κB complexes, nuclear extracts were preincubated with antibodies against the p65 and p50 NF-κB proteins and analyzed by supershift assays. As shown in figure 2B and 2D, the 20(OH)D3-induced complex contains both p50 and p65 proteins. The specificity of the binding to the κB probe was determined by incubating nuclear extract with excess unlabeled (cold) NF-κB oligonucleotide. Since excess unlabeled NF-κB oligonucleotide competed out DNA binding to the κB probe, NF-κB binding was considered specific.

### 20-Hydroxycholecalciferol inhibits NFκB-driven reporter gene activity in keratinocytes

**Figure 3:**
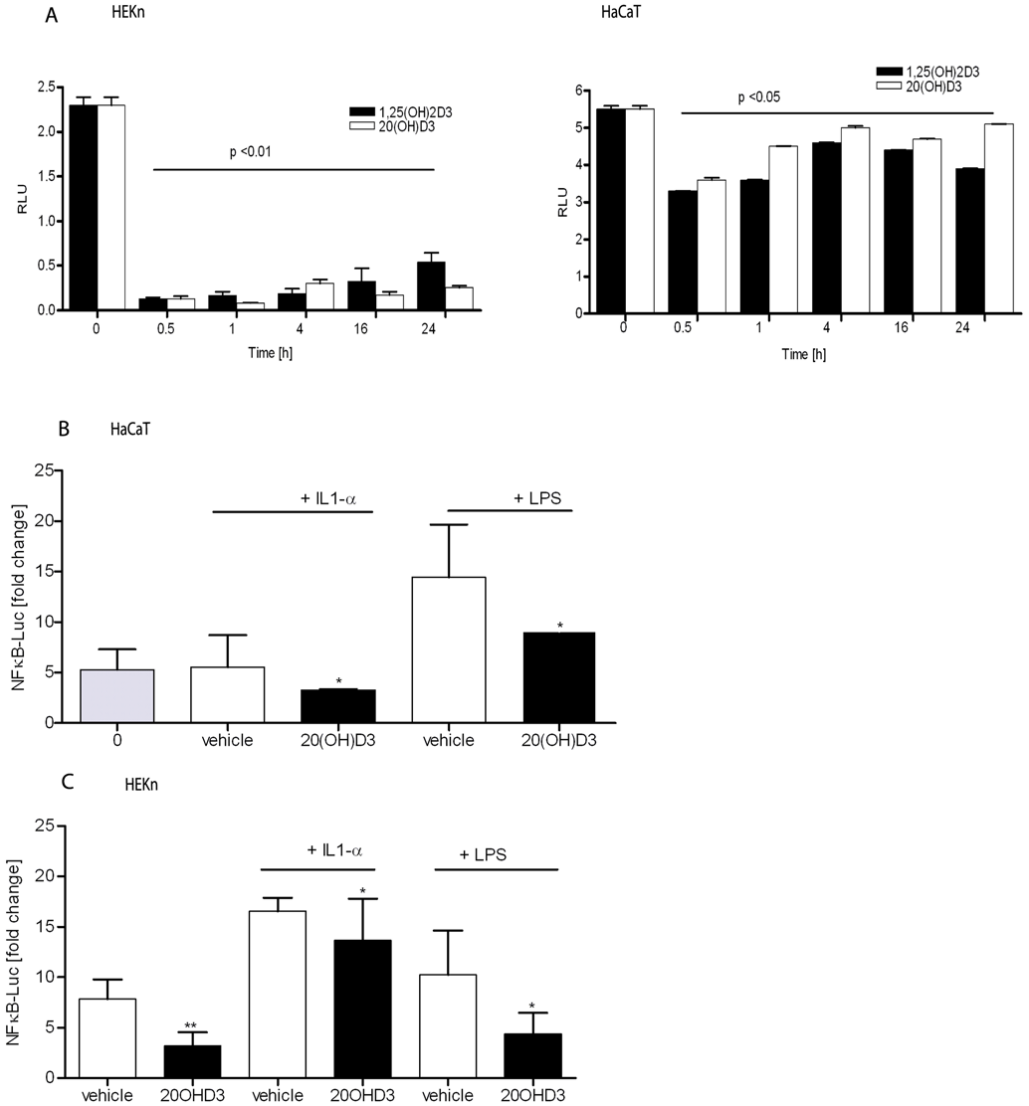
20(OH)D3 treatment inhibits the activation of NFκB-dependant activity in keratinocytes. Keratinocytes were transiently transfected with a NFκB-Luc construct for 24 h then treated with 100 nM 20(OH)D3, 1,25(OH)_2_D3 or ethanol as a vehicle for the indicated time periods (A), or additionally stimulated with LPS (1 µg/ml) or IL-1α (10 ng/ml) for 30 min (B and C, respectively). Cell lysates prepared from HaCaT and normal human keratinocytes were assayed for luciferase activity. The data from six experiments performed in quadruplicate are presented as means±STDEV. *p<0.05 and **p<0.01 between control (non treated cells) and treated cells.

In order to determine functional consequences of the decreased NF-κB DNA binding activity in the keratinocytes treated with 20(OH)D3, we performed gene reporter assays to determine NF-κB driven transcriptional activity (Fig. 3). HaCaT and normal human keratinocytes were transiently transfected with the pNFκB-Luc construct, which contained the firefly luciferase reporter gene driven by NF-κB. In the presence of 1,25(OH)_2_D3 or 20(OH)D3, basal luciferase activity decreased (Fig. 3). The inhibitory effect was more pronounced in normal human keratinocytes, with approximately a 2.5-fold decrease in the reporter activity (p<0.01) (Fig. 3A). In immortalized keratinocytes (HaCaT) the decrease in activity was less pronounced, but was also statistically significant (p<0.05) (Fig. 3A). 20(OH)D3 and 1,25(OH)_2_D3 had similar potency in inhibiting the NF-κB driven reporter in keratinocytes. Interestingly, NF-κB activity was significantly inhibited even after 24 hours of treatment with either agent (Fig. 3A).

To further characterize the inhibitory activity of 20(OH)D3 and 1,25(OH)_2_D3, NF-κB activity in HaCaT and normal human keratinocytes was stimulated by two agents known to induce NF-κB activity, LPS or IL-1α. As shown in Figure 3B, both LPS and IL-1α increased NF-κB-driven luciferase activity in HaCaT cells and normal human keratinocytes, as compared to cells treated with vehicle (<0.01% ethanol). We next examined the effects of 20(OH)D3 or 1,25(OH)_2_D3 on luciferase activity in HaCaT cells stimulated with LPS or IL-1α. Treatment with 20(OH)D3 or 1,25(OH)_2_D3 resulted in a statistically significant (p<0.05) decrease in NF-κB-driven luciferase expression in HaCaT cells stimulated by LPS or IL-1α with 20(OH)D3 and 1,25(OH)_2_D3 exhibiting similar potencies in inhibiting NF-κB activity. We than analyzed luciferase activity in cell extracts from human epidermal keratinocytes (HEKn), treated with 20(OH)D3 or 1,25(OH)_2_D3 and stimulated with LPS or IL-1α. Interestingly, the inhibition by 20(OH)D3 or 1,25(OH)_2_D3 of NF-κB activity was greater when the keratinocytes were stimulated with LPS as compared to IL-1α. 20(OH)D3 was slightly less potent in inhibiting NF-κB activity in keratinocytes when compared to 1,25(OH)_2_D3. Thus, despite the cell-type differences in the stimulation of NF-κB-dependent transcription activity by LPS versus IL-1α, 20(OH)D3 and 1,25(OH)_2_D3 inhibited NF-κB-dependent transcription.

### 20-Hydroxycholecalciferol inhibits translocation of the p65 NFκB protein induced by IL-1α in keratinocytes

**Figure 4:**
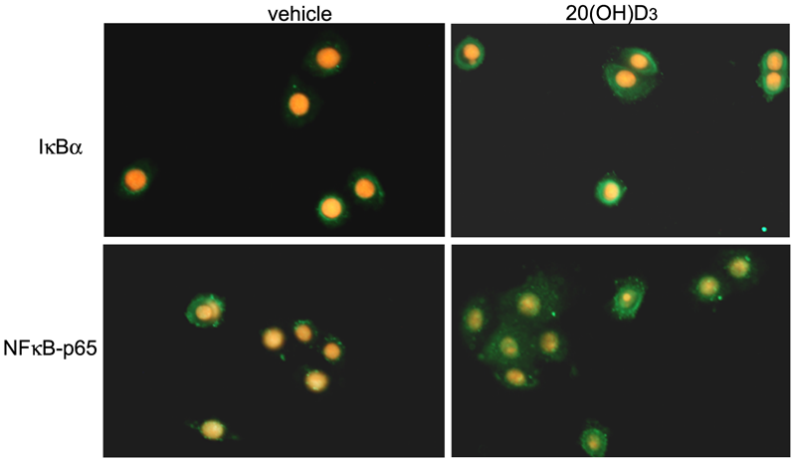
20(OH)D3 inhibits the translocation of NFκB-p65 complex into the nucleus and increases the expression of IκBα in the cytosol of keratinocytes. Primary human keratinocytes, third passage, were incubated for 4 h in KBM medium containing KGF with 100 nM 20(OH)D3 or ethanol vehicle, stimulated with IL-1α for 30 min and then fixed. Cells were stained with anti IκBα or NFκB-p65 antibody, followed by secondary antibody linked to FITC. Nuclei were stained red with propidium iodide. Cells were analyzed using fluorescent microscope at 40× magnification.

To further characterize the inhibitory effect of 20(OH)D3 on NF-κB activity, we examined the cellular localization of the p65 NF-κB protein and the IκBα inhibitory protein in keratinocytes by fluorescent microscopy. As is usually observed in unstimulated cells, NF-κB is localized mainly in the cytoplasm with only minor nuclear staining detected. Stimulation of cells by IL-1α induced p65 translocation from the cytoplasm into the nucleus, indicative of the NF-κB activation. In contrast, treatment of cells with 20(OH)D3 nearly completely blocked the nuclear translocation of p65. In addition, there was a detectable increase in IκBα protein localized in the cytoplasm after treatment with 20(OH)D3 in comparison to vehicle-treated cells (Fig. 4). Similar results were obtained when HaCaT and normal keratinocytes were treated with 20(OH)D3 for 1, 4 or 24 hours (data not shown).

### 20-hydroxycholecalciferol increases IκBα protein levels in keratinocytes

**Figure 5:**
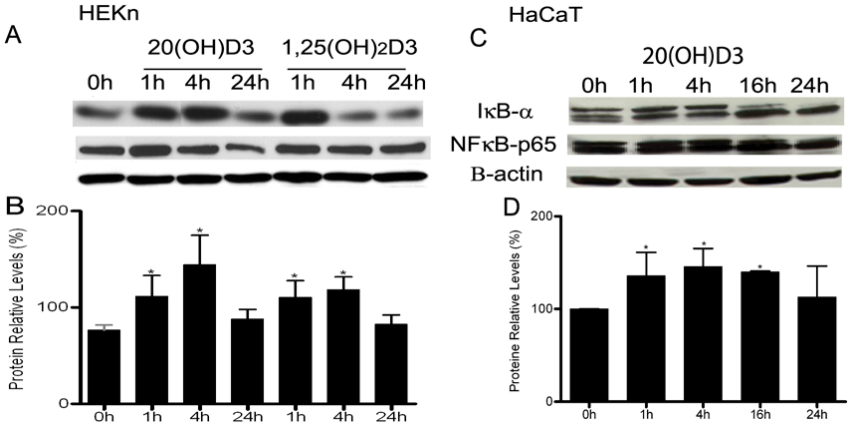
20(OH)D3 increases IκBα protein concentration in keratinocytes and has no effect on NFκB-p65. Keratinocytes, HEKn and HaCaT were stimulated for the indicated times with 100 nM 20(OH)D3, and 100 nM 1,25(OH)_2_D3 (HEKn keratinocytes). Cells were lysed, whole cell extracts prepared, and equivalent amounts of protein were loaded onto polyacrylamide gels. Membranes were incubated with either anti-IκBα, anti-NFκB-p65 or anti β-actin (internal control) (A, C). Protein concentration expressed relative to β-actin was significantly different to the zero-time control for IκBα (p<0.05)(B, D). Results from three separate experiments are expressed as mean±STDEV.

Since we demonstrated by various assays (EMSA, gene reporter assays, and immunofluorescence assays) that 20(OH)D3 inhibits NF-κB activity, we next examined the underlying mechanism responsible for this activity. In the classical NF-κB pathway, NF-κB activity is sequestered it in the cytoplasm by forming a complex with inhibitory IκB proteins. Moreover, as shown in figure 4 20(OH)D3 appears to increase cellular IκB levels as determined by immunofluorescent staining. To determine whether 20(OH)D3 affects the classical NF-κB pathway, the cellular levels of IκBα and the p65 NFκB were determined at various times after 20(OH)D3 addition to cells. 20(OH)D3 induced a time-dependent increase in IκBα levels in whole cell extracts of HEKn (Fig. 5A) and HaCaT keratinocytes (Fig. 5C). IκBα was increased within 1 hour of 20(OH)D3 treatment, and by 16 hours IκBα was diminished to the levels observed in untreated cells. Similar results were obtained when cells were treated with 1,25(OH)_2_D3. In contrast, cellular levels of p65 was unaffected by 20(OH)D3 treatment of keratinocytes. As shown figure 5B and 5D, statistically significant changes were observed for IκBα levels induced by 20(OH)D3 and 1,25(OH)_2_D3 expressed relative to β-actin (p<0.05).

**Figure 6:**
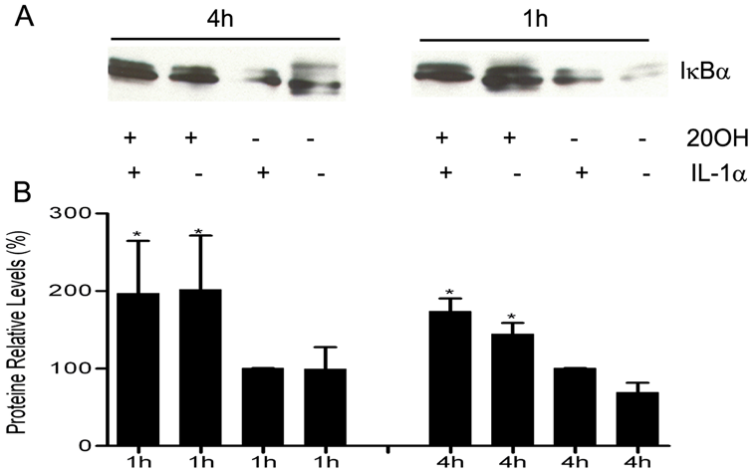
20(OH)D3 increases IκBα protein concentration in cytosolic extracts in keratinocytes. Normal keratinocytes were treated with 100 nM 20(OH)D3 for 1 h and 4 h and then stimulated with or without IL-1α (10 ng/ml) for 30 min. Cells were lysed in lysis buffer and cytosolic extracts prepared. An equal amount of proteins was loaded onto the polyacrylamide gel. Membranes were incubated with antibodies: anti-IκBα and anti β-actin (internal control) (A). Protein concentration expressed relative to the concentration of β-actin is shown in (B). Results are expressed as mean±STDEV.

To further characterize the ability of 20(OH)D3 to inhibit NF-κB activity we stimulated NF-κB activity in normal human keratinocytes with IL-1α and determined IκBα levels in cytosolic extracts. We found that the concentration of IκBα levels were increased after treatment with 20(OH)D3 for 1 and 4 hours (Fig. 6A). Treatment of cells with 20(OH)D3 without IL-1α stimulation had a similar effect on IκBα levels. As shown figure 6B, statistically significant changes were observed for IκBα levels induced by 20(OH)D3 expressed relative to β-actin (p<0.05).

### 20-Hydroxycholecalciferol stimulates IκBα mRNA expression, but does not affect NF- κB mRNA expression in keratinocytes

**Figure 7:**
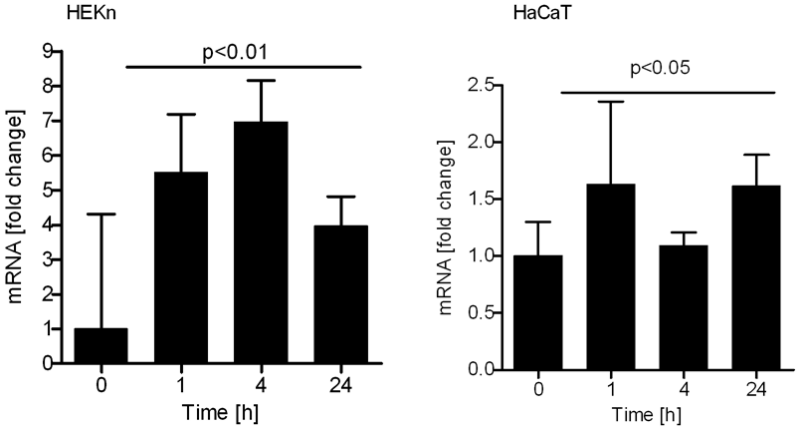
20(OH)D3 increases mRNA levels of IκBα in keratinocytes. HEKn and HaCaT keratinocytes were treated with 100 nM 20(OH)D3 or 1,25(OH)_2_D3 or vehicle for the indicated period of time. Cells were then lysed and total RNA extracted. mRNA levels for IκBα were measured using reagents for RTPCR according to the manufacturer's protocol (Roche Applied Science, Manheim, Germany) and normalized relative to Cyclophylin B RNA. Data are presented as mean±STD (n = 3) *p<0.05 versus control, or **p<0.01 versus control. Time response in the expression of mRNA was shown for NFκBI (IκBα) in both cells.

To determine whether the increased IκBα protein levels in cells treated with 20(OH)D3 resulting from increased IκBα mRNA expression, we measured IκB mRNA levels by quantitative real time PCR (qPCR). As shown in figure 7 the IκBα-mRNA levels were significantly increased after 20(OH)D3 treatment of HaCaT and normal human keratinocytes. The induction by 20(OH)D3 of IκBα mRNA expression was greater in HaCaT cells than in normal keratinocytes. The effect was already detected 1 hour after treatment and returned to basal levels by 24 hours in normal keratinocytes, while the induction of IκBα mRNA persisted up to 24 hours in HaCaT cells. Moreover, the effects of 1,25(OH)_2_D3 on IκBα mRNA levels were qualitatively similar to those noted for 20(OH)D3. In contrast, mRNA levels of the p50 and p65 NF-κB subunits were unaffected by treatment with either 1,25(OH)_2_D3 or 20OHD3 (data not shown).

### 20-hydroxycholecalciferol requires VDR expression for its action on the NF-κB pathway in keratinocytes

**Figure 8:**
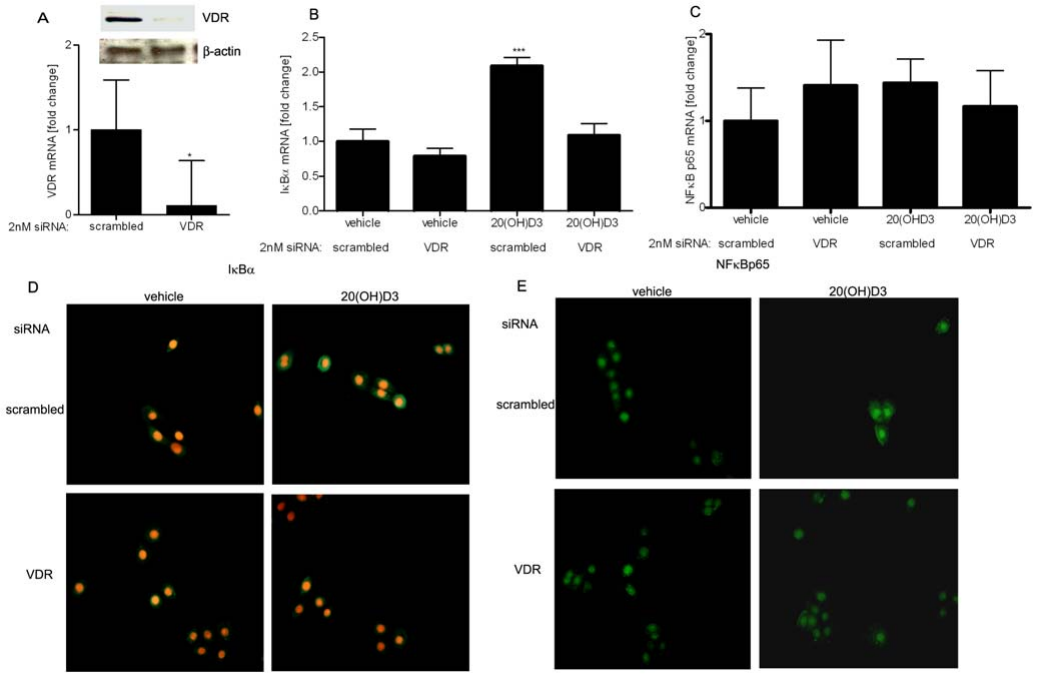
Silencing of the VDR in human keratinocytes attenuates the effects of 20(OH)D3 on IκBα or NFκB p65 (RelA) expression and intracellular transolaction. Keratinocytes were transfected with 2 nM scrambled or VDR siRNA and incubated with 100 nM 20(OH)D3 or vehicle (ethanol) for 4 h. Cells were lysed after treatment and total RNA extracted. VDR mRNA (A), IκBα mRNA (B) and NFκB p65 (RelA) (C) levels were measured using reagents for RTPCR according to manufacturer's protocol (Roche Applied Science, Manheim, Germany) and normalized relative to Cyclophylin B RNA. Data are presented as mean±STD (n = 3) *p<0.05 versus control, or **p<0.01 versus control. Levels of VDR and β-actin were assessed 24 h after transfection with VDR or scrambled siRNA, by western blotting of whole-cell extracts (A). 24 h after transfection with 2 nM scrambled or VDR siRNA, primary human keratinocytes were also incubated for 4 h in KBM medium containing KGF with 100 nM 20(OH)D3 or ethanol (vehicle), then fixed. Cells were stained with anti-IκBα antibody (green), followed by secondary antibody linked to FITC. Nuclei were stained red with propidium iodide (D). Another set of cells was stained with anti-p65 (green) (E). Cells were analyzed using a fluorescent microscope at 20× magnification.

We previously demonstrated that the action of 20(OH)D3 on human keratinocytes is dependent on VDR expression [15]. Therefore, we examined whether the effect of 20(OH)D3 on the NF-κB pathway was also VDR-dependent. Human keratinocytes were transiently transfected with siRNA to knock-down VDR expression, treated with 20(OH)D3 or vehicle (ethanol) and RNA isolated for gene expression analysis by qPCR In parallel experiments cell extracts were analyzed for protein expression by western blot. As shown in figure 8A transfection of keratinocytes with VDR siRNA knocked-down the levels of the vitamin D receptor by approximately ∼80%. Most importantly, knock-down of the VDR in keratinocytes completely blocked IκBα mRNA induction by 20(OH)D3 treatment. In contrast, 20(OH)D3 treatment induced a ∼2 fold increase in IκBα mRNA in cells transfected with scrambled siRNA (Fig. 8B). The mRNA levels for p50 and p65 NF-κB proteins were unaffected by VDR knockdown (data not shown for p50). To further test the ability of VDR knockdown on NFκB translocation, we transfected cells with scrambled or VDR siRNA and treated them with 20(OH)D3, and than examined the cellular localization of the p65 NF-κB protein and the IκBα inhibitory protein in keratinocytes by fluorescent microscopy. In summary, significantly less IκBα protein was localized in the cytoplasm after 20(OH)D3 treatment of VDR knockdown cells as compared with scrambled siRNA-treansfected cells (Fig. 8D). 20(OH)D3 treatment of cells nearly completely blocked the nuclear translocation of p65. In contrast, 20(OH)D3 treatment of VDR knockdown cells did not block the nuclear translocation of p65 (Figure 8E).

## Discussion

We have previously shown that 20(OH)D3 is a product of vitamin D3 metabolism by cytochrome P450scc (see Fig. 1) [7], [10]. Moreover, 20(OH)D3 has significant biological activity in human keratinocytes, as it inhibits their proliferation and stimulates their differentiation [15]. In the present study we demonstrate that 20(OH)D3 is a potent inhibitor of NF-κB activity. Moreover, 20(OH)D3 treatment also increases IκBα protein levels through induction of IκBα mRNA expression. IκBα induction by 20(OH)D3 requires VDR expression, indicating that 20(OH)D3 acts through the classical vitamin D and NFκB regulatory pathways.

The inhibitory effect of 20(OH)D3 on NF-κB activity in keratinocytes was shown by several complementary approaches including NF-κB dependent DNA binding assays, NFκB-driven reporter gene activity assays, as well as western blotting and immunofluorescence analysis of the translocation of p65 NF-κB subunit from cytoplasm into the nucleus. The inhibitory effect of 20(OH)D3 was rapid (within 30 minutes), reached a maximum by 4 hr after addition, and remained detectable as long as 24 hr after addition. The inhibitory effect of 20(OH)D3 on NF-κB-dependent transcriptional activity by luciferase reporter-gene analysis (Fig. 3) paralleled the time course of 20(OH)D3 inhibition of NF-κB-dependent DNA binding activity by EMSA (Fig. 2). The inhibitory effect of 20(OH)D3 on NF-κB was greater in normal human keratinocytes as compared to the effect in HaCaT keratinocytes. This discrepancy might be secondary to the immortalization of HaCaT cells, which might render them less sensitive to 20(OH)D3 treatment. Moreover, 20(OH)D3 had similar potency to that of the well characterized 1,25(OH)_2_D3 in inhibiting NF-κB activity in keratinocytes (no statistically significant difference). The vitamin D analogs, 1,25(OH)_2_D3 and 1,24(OH)_2_D3, have been previously reported to inhibit NF-κB activity [57]. Also, 1,25(OH)_2_D3 has been previously shown to regulate NF-κB DNA binding activity in human keratinocytes through an increase in IκBα expression [55]. In this study, 1,25(OH)_2_D3 inhibited NF-κB binding to the IL-8 κB binding sequence more potently than binding to the p53 κB binding sequence. This selectivity may be mediated through an increased IκBα expression, indicating that vitamin D analogues may exert their immunomodulatory effects through NF-κB regulated proinflammatory cytokines and chemokines. In our study we tested the effect of the novel analog of vitamin D3, 20(OH)D3, not only on NF-κB activity, but also on protein and mRNA levels, as well as the role of VDR in the effect of 20(OH)D3 on NF-κB. We clearly demonstrate that 20(OH)D3 inhibits NF-κB activity with potency similar to that of calcitriol (1,25(OH)_2_D3, the endogenous active form of vitamin D3). The mechanism of action or 20(OH)D3 appears to be very similar to that of 1,25(OH)_2_D3. The hydroxyl group of 20(OH)D3 is attached at the C20 position [7]; which is interesting since the attachment at C1 is considered to be required for full biological activity and calcemic effects [2], [5].

To further characterize the action of 20(OH)D3 on inhibiting NF-κB activity we used known stimulators of the NF-κB pathway, LPS and IL-1α [64]. Keratinocytes can produce a plethora of cytokines including interleukin (IL)-1 and tumor necrosis factor α (TNF) (reviewed in [37]). IL-1 activates keratinocytes and promotes their proliferation and migration. Also, LPS is considered as a potent NF-κB stimulator [65]. In the present study, 20(OH)D3 was found to attenuate NF-κB transcriptional activity induced by both LPS or IL-1 in HaCaT cells and primary keratinocytes, and exhibited a similar potency to 1,25(OH)_2_D3. Interestingly, in normal keratinocytes the inhibition of NF-κB activity stimulated with IL-1α by vitamin D3 hydroxyderivatives was less pronounced then the inhibition of activity stimulated with LPS. This indicates that immortalization changes the responsiveness of keratinocytes to various stimuli, as it has been demonstrated in case of neuropeptides [63], [66]. Nonetheless, both 20(OH)D3 and 1,25(OH)_2_D3 inhibit NF-κB activity in keratinocytes induced by recognized proinflammatory stimuli.

Stimulation of NF-κB activity by LPS and IL-1α does not alter the action of 20(OH)D3 on NF-κB activity, since 20(OH)D3 treatment of cells with or without exogenous stimulation had similar effects on p65 localization and IκBα levels. Although NF-κB can be activated through both classical and alternative signaling pathways, previous studies have indicated that IL-1 and LPS activate NF-κB through the classical signaling pathway [46]. In this general pathway, p50∶p65 dimers are sequestered in the cytoplasm by IκB proteins. LPS and IL-1 stimulate IκB kinase activity, resulting in the subsequent IκB phosphorylation and ubiquitinylation. Then IκB is targeted for proteosomal degradation, which allows p50∶p65 dimers to translocate to the nucleus, bind to DNA and activate the transcription of NF-κB-dependent genes. Consistent with this general pathway we show that both LPS and IL-1 stimulate NF-κB transcriptional activity as well as result in IκB degradation. Most importantly, we show that 20(OH)D3 acts as an immunosuppressive agent in human keratinocytes by blocking the activation of this signaling pathway by both IL-1 and LPS. 20(OH)D3 not only inhibits the translocation of the p65 NF-κB protein from cytoplasm to nucleus in keratinocytes, but also increases the cellular levels of the inhibitory NF-κB protein, IκB, thus sequestering the NF-κB in the cytoplasm as transcriptionally inactive NF-κB/IκB complexes. Since recent studies demonstrate that activation of the alternative NF-κB pathway can also lead to the translocation of p65-containing dimers into the nucleus [42], our data cannot exclude the possibility that 20(OH)D3 also blocks this signaling pathway as well. Detailed analysis of the alternative signaling pathway will be the subject of future studies.

In previous studies we showed that the action of 20(OH)D3 on proliferation and differentiation in keratinocytes requires VDR expression [15]. In the present study we find that silencing VDR expression in keratinocytes blocks the inhibitory actions of 20(OH)D3 on NF-κB activity (Fig. 8). Therefore, our data indicates that both 20(OH)D3 (novel ligand) and 1,25(OH)_2_D3 (classical ligand) suppress NF-κB activity through a VDR-mediated signaling pathway. Although the mechanism of this pathway in inhibiting NF-κB activity requires more in-depth analysis, our studies demonstrate that 20(OH)D3 can induce anti-inflammatory actions similar to those mediated by calcitriol (1,25(OH)_2_D3) via the VDR-mediated inhibition of NF-κB activity. Interestingly vitamin D analogues are now widely used drugs for the treatment of psoriasis, an inflammatory and hyperproliferative dermatoses (reviewed in [49]). Therefore, we believe that 20(OH)D3 holds promise as a novel therapeutic agent in the prevention and therapy of inflammatory, auto-immune and hyperproliferative skin diseases.

**Figure 9:**
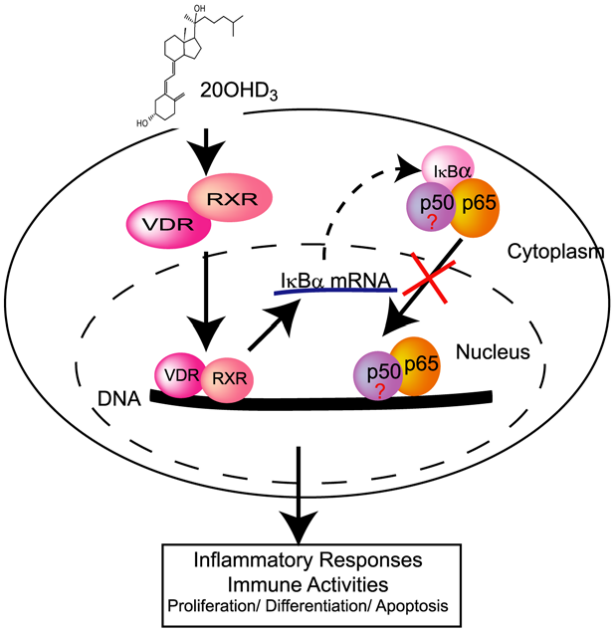
A schematic representation of the effects of 20(OH)D3 on NFκB signal transduction in keratinocytes.

Recently, new and important immunomodulatory effects of vitamin D analogs have been characterized, especially those for 1,25(OH)_2_D3 [1], [25], [67]. Inhibitors targeting the NF-κB signaling pathway effectively suppress NF-κB activity, protect and relieve inflammatory symptoms, and induce apoptosis of tumor cells. NF-κB represents an attractive drug target for therapy of inflammatory and autoimmune disorders, as well as for cancer. Thus, 20(OH)D3 is a new powerful analog of vitamin D3 that is produced by enzymatic activity of CYP11A1 [7], [10], and have pleiotropic activities through its ability to modulate the NF-κB signaling pathway as illustrated in figure 9. Increased expression of IκBα and inhibition of NF-κB activity in keratinocytes induced by 20(OH)D3 may be one mechanism by which this (potentially endogenous) vitamin D analog could exert beneficial effects in inflammatory and auto-immune disorders.

## Materials and Methods

### Cell culture

Immortalized human keratinocytes (HaCaT), which are frequently used for studies on biological effect of 1,25(OH)_2_D3 [30], [68], were cultured in Dulbecco's Modified Eagle Medium supplemented with glucose, L-glutamine, pyridoxine hydrochloride (Cell Grow), 5% fetal bovine serum (Sigma) and 1% penicillin/streptomycin/amphotericin antibiotic solution (Sigma) [6]. In order to eliminate potential interference by sterols present in the serum [69], 5% charcoal/dextran-treated bovine serum (HyClone) was used to test the effects of active forms of vitamin D. In addition, cells were serum-deprived for 24 h before treatment. Normal human epidermal keratinocytes were isolated from neonatal foreskin (HEKn) and grown in KGM medium supplemented with KGF (Lonza) on collagen-coated plates [15]. For experiments cells in their third passage were used.

### Immunofluorescent staining

HEKn cells were seeded onto cover glasses in 6-well plate and treated with 100 nM of 20(OH)D3 for 24 h. Control cells were treated with solvent (<0.1% ethanol). After treatment cells were washed in PBS and fixed in 4% paraformaldehyde. Cells were than incubated in permeabilizing solution (0.2% Triton-X 100 in PBS) for 5 min, washed with PBS and blocked in 2% BSA for 30 min. Primary antibody, either goat anti-rabbit-p65 (1∶100) or goat anti-rabbit-IκB (1∶100) in 1% BSA, was added to the cells and incubated overnight at 4°C. After extensive washing in PBS, cells were incubated in the secondary antibody solution comprising goat-anti-rabbit-Alexa Fluor 488 (Invitrogen, 1∶500 in PBS) and incubated for 1 h at room temperature in the dark. Cells on cover glass were washed and mounted with mounting medium containing propidium iodine (Vectashield). Stained cells were analyzed using a fluorescent microscope at 40× magnification.

### Transfection and Reporter assay

The constructs pLuc, pHRLTK and NFκB-Luc have been described previously [70]. According to our protocols [63], [66], HaCaT and normal epidermal keratinocytes were transfected using Lipofectamine Plus (Invitrogen, Carlsbad, CA) in DMEM or KGM medium, with firefly luciferase reporter gene plasmid and with phRL-TK (expresses Renilla luciferase and serves as normalization control; Promega, Madison, WI). At 24 hr after transfection, the medium was changed and the cells were treated with the vitamin D3 derivatives or vehicle (<0.1% ethanol) for 0.5, 1, 4, 16 and 24 h. Following this protocol, cells were also treated with 10 ng/ml interleukin 1α (IL-1α) (Sigma) or 1 µg/ml LPS (Sigma) for 30 minutes. The Firefly luciferase and Renilla luciferase signals were recorded with a TD-20/20 luminometer (Turner Designs, Sunnyvale, CA); background luminescence was subtracted and the resulting promoter specific firefly signal was divided by the Renilla signal (proportional to the number of transfected cells). The values obtained were calculated relative to control (untreated) cells, and expressed as relative fold change.

### siRNA transfection

Keratinocytes were transfected with 2 nM VDR or scrambled siRNA (Dharmacon), on-Target plus smart pool human VDR or on-Target plus siControl non-targeting pool, using lipofectamine plus (Invitrogen) in DMEM medium. Twenty four hours after transfection, cells were treated for an additional 4 h with 100 nM 20(OH)D3 or vehicle (ethanol), mRNA was isolated and used for gene expression analysis or cells were stained for IκBα or NFκB p65 and examined for protein localization by fluorescent microscopy.

### Preparation of cell lysates

Cells were treated with 20(OH)D3 or 1,25(OH)_2_D3, and whole cell lysates were prepared as described previously [71], [72]. Cells were resuspended in RIPA buffer containing protease inhibitor cocktail (Sigma) and PMSF. Nuclear extracts were prepared as described previously [73]. In brief, HaCaT or normal human keratinocytes were treated with 100 nM 20(OH)D3 for 0, 0.5, 1, 4, 16 or 24 h, and then stimulated with or without interleukin 1α (10 ng/ml) for an additional 30 min. The cells were harvested, pelleted and resuspended in STM buffer (20 mM Tris-HCl, 250 mM sucrose and 1.1 mM MgCl_2_). The nuclear pellet was resuspended in 30 µl nuclear extraction buffer containing 0.4 M KCl, 5 mM 2-mercaptoethanol and protease inhibitor cocktail (1∶100 dilution, Sigma) in STM buffer, and incubated on ice for 30 min with intermittent shaking and then centrifuged at 14,000×g for 20 min at 4°C. The protein content in the supernatant was quantified using the Bradford protein assay kit [74]. Cytosolic extracts were prepared as described previously with minor modifications [55]. Cell pellets were resuspended in hypotonic buffer (10 mM HEPES pH 7.9, 10 mM KCl, 0.2 mM EDTA, 0.1 mM EGTA, 1 mM DTT, 0.5% NP40, protease inhibitor cocktail (Sigma) and 20 mM PMSF), and after a 20 min of incubation on ice the suspension was centrifuged at 4°C for 10 minutes at 5,000×g. The centrifuged supernatant was considered the cytosolic extract.

The levels of IκBα, the p65 NF-κB protein, and β-actin were assessed by immunoblotting. Primary antibodies used were the rabbit polyclonal antibodies directed against IκB-α (Santa Cruz, 1∶500 dilution); p65 (Santa Cruz, 1∶500 dilution) and β-actin-peroxidase (Sigma, 1∶5000 dilution). The secondary antibody used was anti rabbit IgG (Santa Cruz, 1∶7,000 dilution) and anti-mouse IgG (Santa Cruz, 1∶5,000) conjugated to horseradish peroxidase. Signals were detected using ECL kit Supersignal West Pico Chemiluminescent Substrate (Pierce). The intensity of bands was measured using ImageJ Software. Results for whole cell and cytosolic protein levels were expressed relative to β-actin levels [74]. Levels of VDR and β-actin 24 h after VDR siRNA transfection were assessed in western blots with (VDR(D-6)) antibody (Santa Cruz, 1∶400).

### Electrophoretic mobility shift assay (EMSA)

DNA binding activity was determined by EMSA using a consensus NF-κB IRDye-labeled oligonucleotide probe (LI-COR). The DNA binding reaction consisted of 2.5 to 5 µg of the nuclear extract, the NF-κB probe and gel shift binding buffer containing of 2.5 mM DTT, 0.25% Tween-20 and 0.25 mg/ml poly(dI) ∶poly(dC). The reaction was carried out at room temperature in the dark for 30 min. For supershift assays 1 µg of p65 or p50 antibody (gift of the NCI Preclinical Repository) was added to the nuclear extract prior to DNA binding and incubated for 30 min at 4°C. Orange loading dye was added to samples which were loaded on pre-run 5% TBE gels and run at 70 V for 2 h. The gel was scanned using an Odyssey Infrared Imaging System (LI-COR, Inc,. Lincoln, NE).

### Real-time RT PCR

The RNA from HaCaT and normal keratinocytes treated with 20(OH)D3 or 1,25(OH)_2_D3 was isolated using Absolutely RNA Miniprep Kit (Stratagen). Reverse transcription (100 ng/reaction) was performed with Transcriptor First Strand cDNA Synthesis Kit (Roche). Real-time PCR was performed using cDNA diluted 10-fold in sterile water and a TaqMan PCR Master Mix (n = 3). Reactions were performed at 50 °C for 2 min, 95 °C for 10 min and than 50 cycles of 95 °C for 15 s, 60 °C for 1 min). The primers and probes were designed with universal probe library (Roche): IκBα primers (left: GTCAAGGAGCTGCAGGAGAT and right: GATGGCCAAGTGCAGGAA), probe #38; NFκB1 primers (left: ACCCTGACCTTGCCTATTTG and right: AGCTCTTTTTCCCGATCTCC), probe #39; and RelA primers (left: CGGGATGGCTTCTATGAGG and right: CTCCAGGTCCCGCTTCTT), probe #47; VDR primers (left: CTTACCTGCCCCCTGCTC and right AGGGTCAGGCAGGGAAGT), probe #58. The data was collected on a Roche Light Cycler 480. The amounts of product were compared to Cyclophilin B using a comparative C_T_ method.

### Statistical analysis

Data are presented as means±STDEV and were analyzed with Student's t-test (for 2 groups) and one-way Anova with appropriate post-hoc test (for more than 2 groups) using Excel (Microsoft) and Prism 4.00 (GraphPad Software, San Diego), respectively. Statistically significant differences are denoted with asterisks: *P<0.05, **P<0.001.
